# Dynamic DNA methylation changes during colorectal oncogenesis with insights from adenoma stages

**DOI:** 10.1038/s41598-025-28656-5

**Published:** 2025-11-25

**Authors:** Alexis Overs, Chloé Molimard, Jules Durand, Frédéric Bibeau, Laurent Arnould, Franck Monnien, Claire Clavier, Christophe Borg, Michael Guittaut, Jean-Paul Feugeas, Eric Hervouet, Paul Peixoto, Zohair Selmani

**Affiliations:** 1 Bioinformatique et Big Data Au Service de La Santé, Centre Hospitalier Universitaire de Besançon, 3 Bd Fleming, 25000 Besançon, France; 2 INSERM UMR1098 RIGHT, Université Marie et Louis Pasteur, Besançon, France 25000; 3 Anatomie et cytologie pathologiques, Centre Hospitalier Universitaire de Besançon, 3 Bd Fleming, 25000 Besançon, France; 4 Anatomie et cytologie pathologiques, Centre Georges François Leclerc, 1 Rue du Professeur Marion, 21000 Dijon, France; 5 Oncologie médicale, Centre Hospitalier Universitaire de Besançon, 3 Bd Fleming, 25000 Besançon, France; 6 Oncobiologie, Centre Hospitalier Universitaire de Besançon, 3 Bd Fleming, 25000 Besançon, France

**Keywords:** Colon adenoma, DNA methylation, Dynamic, Epigenetics, Colorectal cancer, Biomarker, Oncogenesis, Tumour biomarkers, Colorectal cancer, Metabolic pathways, DNA methylation

## Abstract

The dynamics of colorectal epigenetics within the adenoma stages of oncogenesis remain undocumented. In this study, we investigated DNA methylation dynamics in colorectal cancer oncogenesis from non-tumor colon tissue to low-grade, high-grade adenoma and adenocarcinoma. The methylome of 12 low-grade and 19 high-grade colorectal adenomas was determined via the EPIC v1 Human Methylation BeadChip. These methylation profiles were complemented with the methylomes of 206 non-tumor colon and 22 colon adenocarcinoma samples from the GEO and TCGA databases. Differentially methylated CpGs were identified via Student’s t test and used to monitor the evolution of the colon methylome during oncogenesis. The differentially methylated promoters were used to infer the associated biological process via gene ontology and the evolution of the methylation of 34 described colorectal cancer DNA methylation biomarkers was explored. A total of 11.9% of the colon methylome was significantly altered (q < 10^− 4^) during oncogenesis, with half corresponding to DNA demethylation. Of which, 67.4% occurred during the transition from non-tumor colon tissue to low-grade adenoma. A total of 9% of the DNA methylation changes were specific to low-grade and/or high-grade adenomas. The biological pathways related to the sensory perception of odor and stimulus were hypomethylated early, nucleic acid metabolic process were methylated early, post-transcriptional regulation were transiently hypomethylated and mitotic cell cycle were transiently methylated. Twenty-one out of 34 the biomarkers were methylated in low-grade adenomas and 11 out of 34 in high-grade adenomas. This suggests that they could be used to distinguish stages of oncogenesis. This study provides insight into the dynamics of colonic epigenetics during oncogenesis, with early DNA methylation changes in low-grade adenomas associated with transient DNA methylation changes. However, the causality of these changes remains to be elucidated. This study also explores the evolution of known biomarkers and their clinical applications for indirectly asserting the tumor’s stage.

**Supplementary Information:**

The online version contains supplementary material available at 10.1038/s41598-025-28656-5.

## Introduction

 Colorectal cancer is a major health problem in high-income countries, being the second leading cause of cancer-related deaths, with more than 900,000 cases reported worldwide in 2022^1^. The oncogenesis of colorectal cancer has been described for most cases as adenocarcinoma arising from colonic dysplasia, from low-grade adenoma to high-grade adenoma to adenocarcinoma^2^. The World Health Organization (WHO) Classification of Tumors of the Digestive System defines conventional colorectal adenoma as a benign, premalignant neoplasm composed of dysplastic epithelium. A two-tiered stratification is used to classify adenomas into low-grade and high-grade adenomas by a pathologist^3^.

Given the central role of adenomas in colorectal oncogenesis, the study of molecular alterations such as DNA methylation is essential to better understand tumor progression and identify potential biomarkers.

Indeed, DNA methylation has emerged as a hallmark of cancer^4^ and a marker of cellular aging that predicts cancer risk in affected tissues^5–8^. DNA methylation has also been shown to vary with anatomic location within the colon and sex^9^.

DNA methylation in colorectal adenomas has been studied previously^10–13^, but few methylome-wide analyses of colorectal oncogenesis have been performed via the Illumina EPIC Methylation Beadchip^14^. The published studies did not differentiate between low-grade and high-grade adenomas^10–14^.

Large-scale DNA methylation changes have been described during colorectal oncogenesis, with global DNA hypomethylation responsible for genomic instability associated with hypermethylation of tumor suppressor gene promoters.

Studying the DNA methylation process may provide a better understanding and identify biomarkers of different stages of colorectal oncogenesis.

The aim of this study was to investigate DNA methylation changes during the early stages of colorectal oncogenesis, with a distinction between low-grade and high-grade adenomas to explore the biological pathways altered during oncogenesis and the evolution of known colorectal cancer biomarkers for clinical applications.

## Methods

### Samples

Thirty-one colon adenomas were obtained from formalin-fixed paraffin-embedded (FFPE) samples from the Tumorothèque régionale de Franche-Comté (Table 1), including twelve low-grade adenomas and nineteen high-grade adenomas.

Grading of adenoma dysplasia was performed via the 2019 WHO Classification of Tumors of the Digestive System, which uses a two-tiered system to distinguish low-grade adenomas from high-grade adenomas. The difference between high-grade and low-grade adenomas was determined by a digestive pathologist by the presence of complex architectural patterns and cytologic features indicative of high-grade dysplasia^3^.

In association with these local samples, non-tumor colon and colon adenocarcinoma methylomes were downloaded from GEO.

The non-tumor colon methylomes from the GSE132804 series (*n* = 206) were generated by Wang et al.^15^, who analyzed the epigenetic aging of 206 colon tissues in detail. Adenocarcinoma methylomes from the GSE149282 series (*n* = 10) were generated by Muhiddin et al.^16^, who performed open chromatin profiling. Twelve additional adenocarcinoma methylomes were obtained from the Human Cancer Models Initiative (HCMI), excluding organoid methylation data.

Three samples, one low-grade adenoma from the Tumorothèque régionale de Franche-Comté and two adenocarcinomas from GSE149282 had a methylated *MLH1* promoter (Supplementary Fig. 1), suggesting MSI status and were therefore excluded from the study.

**Table 1:** Clinical characteristics of the patients.

	CHU of Besançon		HCMI projet		GEO Database				
	Low-grade adenoma *n* = 12	High-grade adenoma *n* = 19	Colonic adenocarcinoma *n* = 12		GSE149282 Colonic adenocarcinoma *n* = 10		GSE132804 Non-tumor colonic tissues *n* = 206	GSE48684 normal colon *n* = 41, adenoma *n* = 42 adenocarcinoma *n* = 64	GSE42921 normal colon *n* = 12
Beadchip	EPICv1	EPICv1	EPICv1		EPICv1		EPICv1	450k	450k
Age									
Mean	74	71.4	66		66.3		59.5	NA	14.7
Min - Max	60–86	46–91	51–76		59–73		19–85	NA	9–17
Stage									
I	n/a	n/a	1		0		n/a	0	n/a
II	n/a	n/a	2		1		n/a	0	n/a
III	n/a	n/a	5		7		n/a	0	n/a
IV	n/a	n/a	2		2		n/a	0	n/a
NA	n/a	n/a	2		0		n/a	64	n/a
Sex									
Male	5	12	9		5		97	58	8
Female	7	7	3		5		109	89	4
Histology									
Adenocarcinoma type NOS	n/a	n/a	12		10		n/a	0	n/a
NA	n/a	n/a	0		0		n/a	64	n/a
Anatomic site									
Right colon	4	10	2		0		0	27	0
Left colon	8	7	7		0		206	39	0
Rectum	0	0	0		0		0	1	0
NA	0	2	3		10		0	80	12

NA: not available, n/a: not applicable.

## DNA methylation assessment

DNA was extracted from FFPE samples via the QIAmp DNA Mini Kit^®^ (Qiagen, Netherlands) according to the manufacturer’s instructions. DNA quantification was performed via a Qubit^®^ fluorometer (Invitrogen, USA). DNA was bisulfited and converted via the Diagenode Premium Bisulfite Kit^®^ according to the manufacturer’s instructions.

Methylomes were obtained via Illumina Methylation EPIC BeadChip v1 and analyzed via R software v4.4.2^17^ and the tidyverse suite^18^, minfi^19^, missMethyl^20^, limma^21^ and InfiniumPurify^22^ packages.

## Bioinformatics analysis

### Methylation values

The idat files were processed into beta values in the same batch via the minfi package. The beta values were then normalized via functional normalization associated with background and dye bias correction^23^. After normalization, the beta values were converted to M values^24^. For a given CpG, the beta value is computed from the light signal of the BeadChips probes and can be interpreted as the percentage of methylation of the CpG. M values are an arithmetic transformation of beta values and are the logarithm of the ratio of methylated to unmethylated intensity. Negative M values are unmethylated (beta value < 0.5), positive M values are methylated (beta value > 0.5), and M values near zero are intermediate (beta value approximately 0.5). Probes on the X and Y chromosomes (*n* = 19090 and 537, respectively) were excluded from the analysis.

## Colonic tissue content

The content of colon tissue was estimated via the getPurity function of the InfiniumPurity package^22^, with non-tumor colon samples used as a reference. The tumor cellularity of the adenocarcinomas and the fraction of dysplastic cells in the adenomas were estimated separately. The getPurity function identifies 1,000 differentially methylated CpGs with high variance between non-tumor and tumor samples. These CpGs were then used to estimate the cellularity of each sample via density evaluation of a Gaussian kernel.

### PCA

Principal component analysis of the methylation data was performed via the R package FactoMineR^25^. The number of principal components was set to 17, and the data were not scaled because of the bimodal distribution of M values.

## Differentially methylated CpGs

Differential methylation analysis was performed via the limma R package. Linear models were fitted to M values with the lmFit function, and empirical Bayes was applied using eBayes to improve variance estimation.

A differentially methylated CpG (DMC) was considered significant if the q value was less than 10^–4^ and if these was an absolute difference in methylation of at least 2 M values.

## Evolution of the methylation status

The evolution of the methylation status was inferred based on the methylation status of different tissue types, assuming an evolution from non-tumor tissue to low-grade adenoma, then to high-grade adenoma, and finally, to adenocarcinoma. “Definitive methylation” changes are defined as modifications in the methylation status between non-tumor colonic tissue and adenocarcinoma. These changes can occur during the transition to low-grade or high-grade adenomas, or during the transition to adenocarcinoma. “Transitory methylation” changes are defined as having the same methylation status between non-tumor tissue and adenocarcinoma, but a different status from low-grade and/or high-grade adenomas.

### Gene ontology

Gene ontology enrichment analysis was performed on the Gene Ontology consortium^26^ via the missMethyl package^20^. The gene lists were generated via the DMC in the promoter region (TSS200, TSS1500 and 1 st exon) compared to the list of genes whose promoters were covered by the methylation BeadChip.

### Validation with 450k BeadChip datasets

Methylation changes were verified with two external GEO series acquired with the Infinium Methylation 450k BeadChip. A total of 41 non-tumor colons, 42 colon adenomas and 64 colon adenocarcinomas were obtained from the GSE48684 series, and 12 non-tumor colons were obtained from the GSE42921 series. These datasets did not discriminate between low-grade and high-grade adenomas. For comparison with the external dataset, low-grade and high-grade adenomas from our dataset were pooled.

## Results

### Methylation changes during colonic oncogenesis

#### Tumor cellularity

**Fig. 1:**
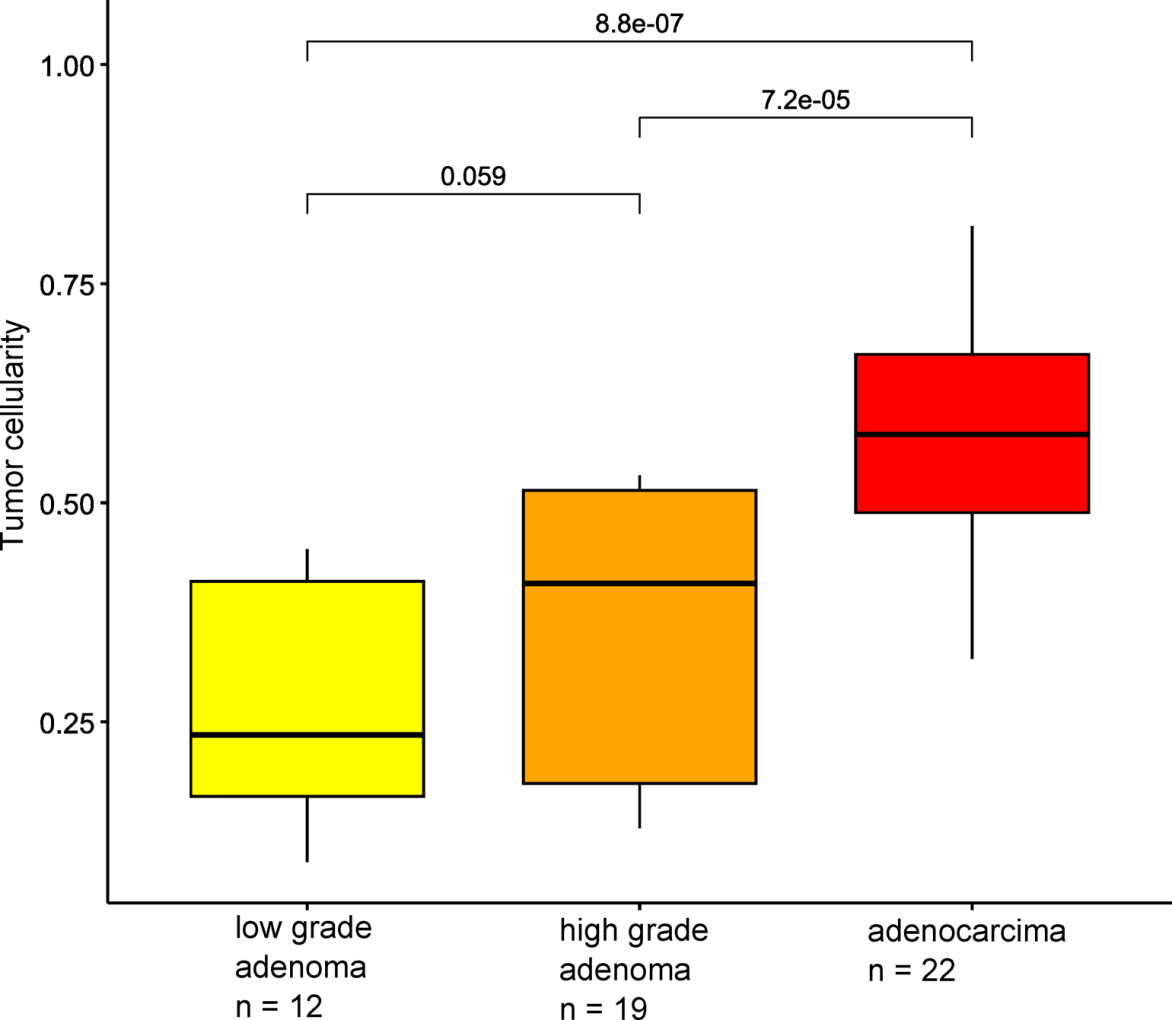
Tumor cellularity according to histologic types.

Tumor cellularity was significantly lower in the adenoma samples than in the adenocarcinoma samples (*p* < 10^− 5^). There were no significant differences between low-grade and high-grade adenomas (*p* = 0.059) (Fig. 1).

The ratio of tumor to non-tumor cellularity was evaluated with the InfinumPurify package. Tumor cellularity was significantly higher in adenocarcinoma samples than in adenoma samples (*p* < 10^− 4^, Wilcoxon test) and no difference was observed between low-grade and high-grade adenomas (*p* = 0.059, *Wilcoxon test).*

### Global distribution

**Fig. 2:**
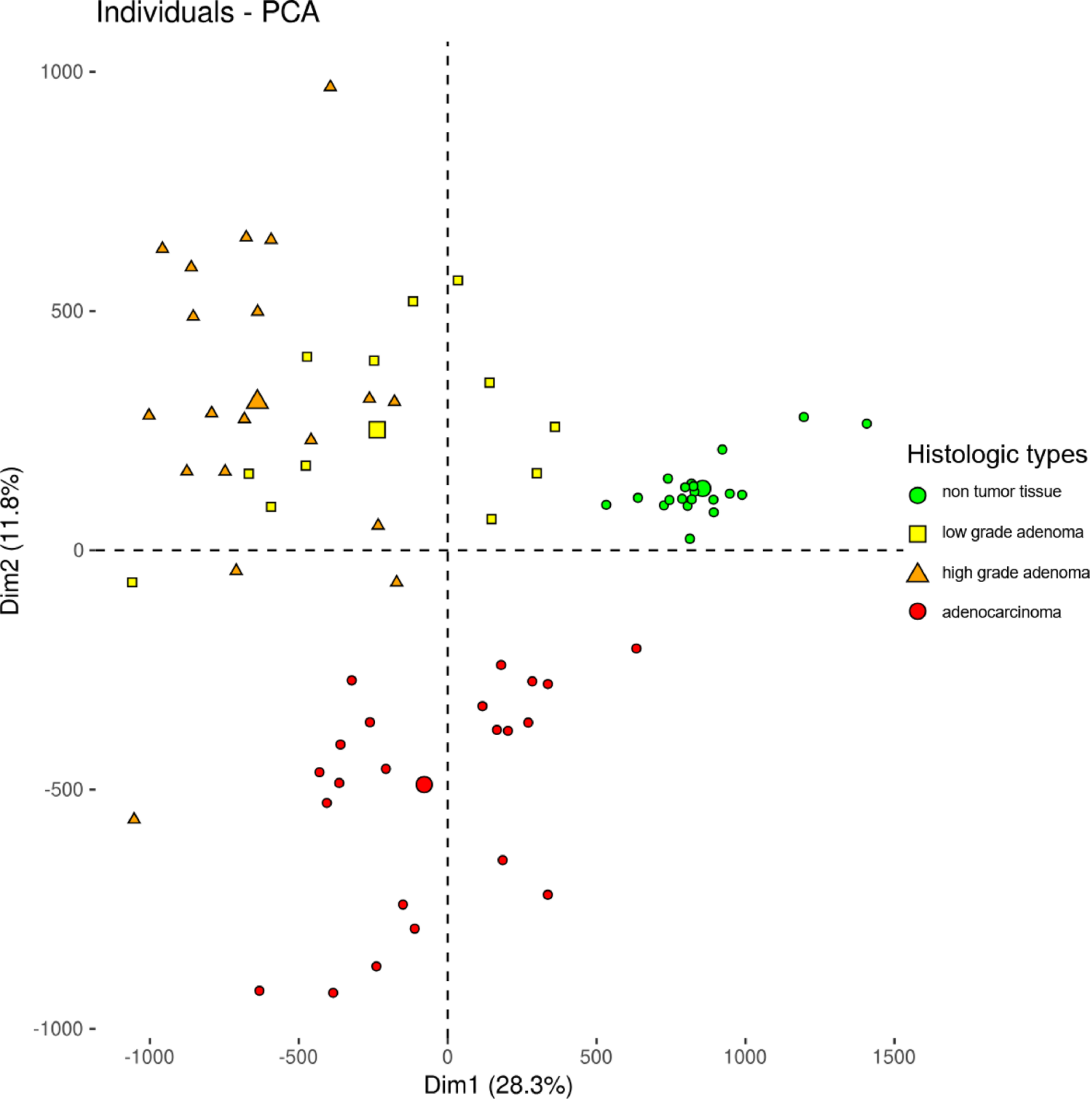
PCA of the samples by methylation data.

The PCA grouped the samples according to their histologic type. The non-tumor group was randomly subsampled (*n* = 20) to match the number of dysplasia and adenocarcinoma samples. The first dimension of the PCA separates non-tumor tissues from adenomas and adenocarcinomas. The second dimension separates adenomas from adenocarcinomas (Fig. 2).

Principal component analysis of non-tumor colonic tissue, adenomas, and adenocarcinomas methylation data revealed segregation of the samples by histology. The non-tumor colonic tissues were randomly downsampled (*n* = 20) to be on the same scale as the number of low-grade adenomas (*n* = 12), high-grade adenomas (*n* = 19) and adenocarcinomas (*n* = 22).

### Major epigenetic changes

**Fig. 3:**
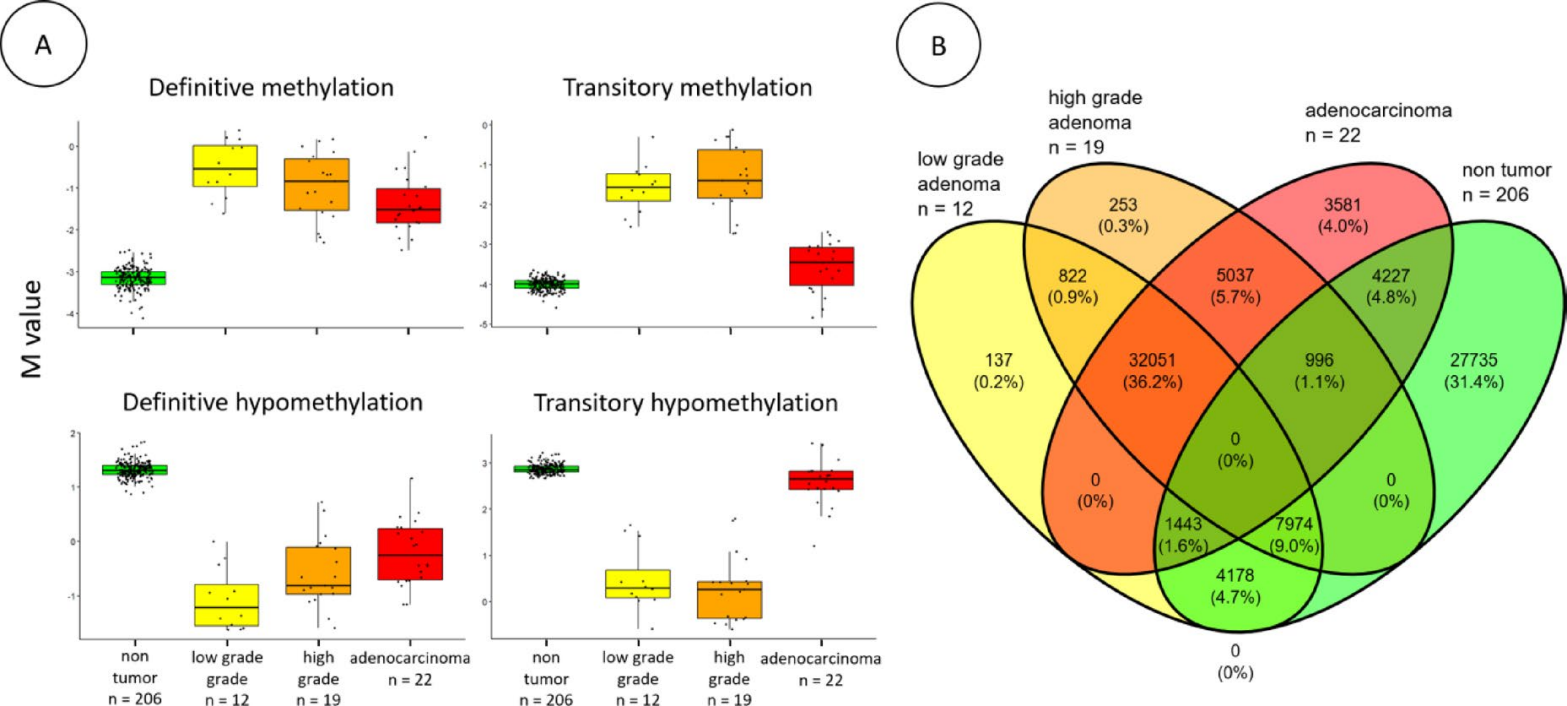
Boxplot and Venn diagram of CpG methylation across the sample types.

Overall, 11.9% of the methylome is significantly modified during oncogenesis (linear models and empirical Bayes, q < 10^− 4^ and an absolute difference of at least 2 M value). The observed changes are equally divided between hypomethylation and methylation. Approximately 67.4% of DNA methylation changes occur during the transition from non-tumor colon tissue to low-grade adenoma (Fig. 3). Methylation mostly occurred in CpG islands and CpG shores and hypomethylation mostly occurred in CpG shelves and open seas (Supplementary Table 1).

However, we observed that some methylation changes are specific to adenoma samples. These adenoma-specific methylation changes are less common than the definitive changes and represent approximately 9% of the methylation changes during oncogenesis.

***A***: Mean M values of differentially methylated CpGs across colon tissue types, separated into four groups based on methylation profile distribution: Definitive methylation (32051 CpGs), Transitory methylation (822 CpGs), Definitive unmethylation (27735 CpGs) and Transitory unmethylation (4227 CpGs). ***B***: Distribution of differentially methylated CpGs according to the linear model and empirical Bayes (q < 10 − 4 and an absolute difference of at least two M value).

### Pathways involved

**Fig. 4:**
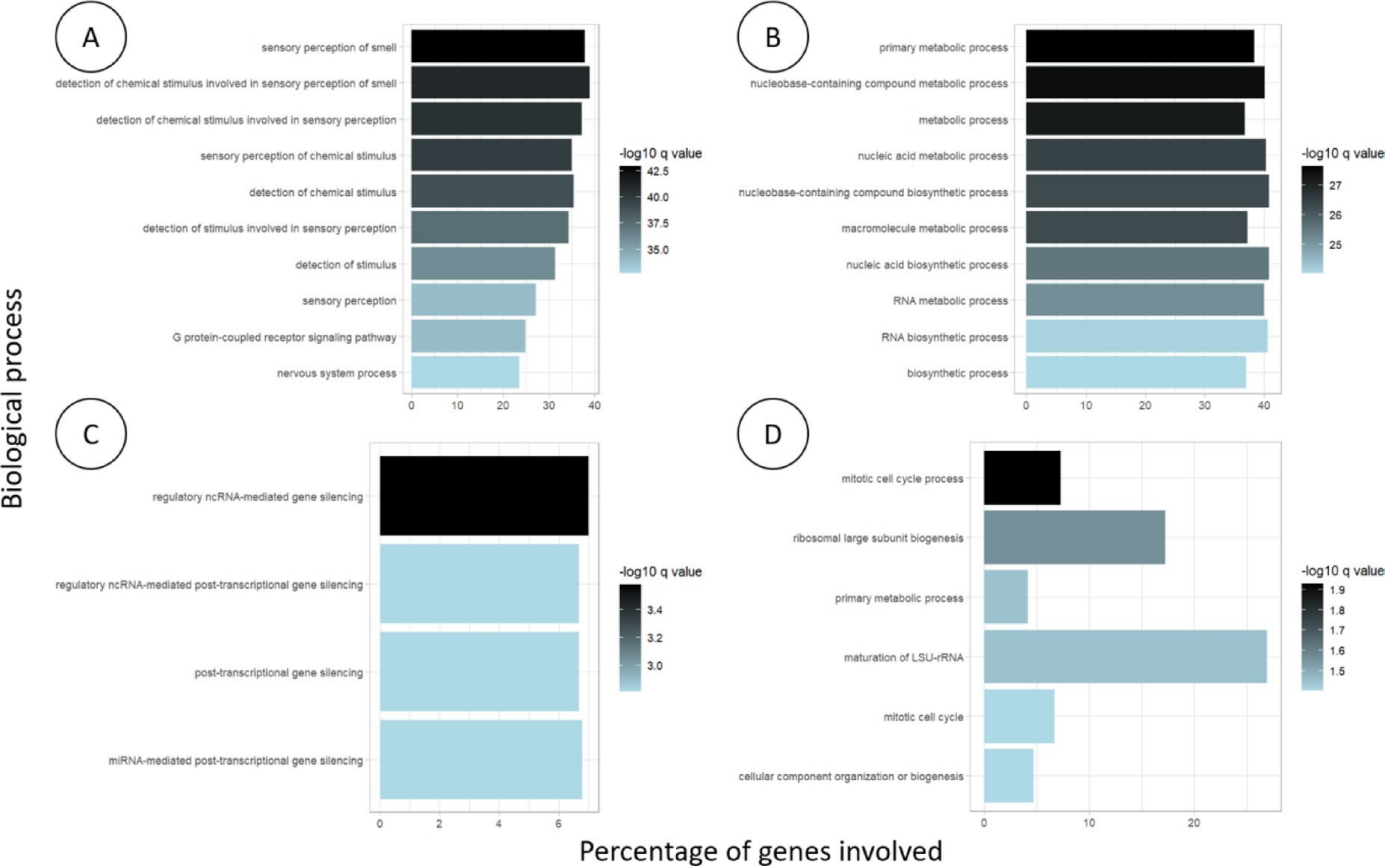
Ontology of biological processes involved in early and transitory methylation changes.

Biological processes involving genes with methylated promoters during oncogenesis were associated with nucleic acids process (2,215 out of 5,492 genes, q < 20^− 44^). Biological processes of genes with unmethylated promoters during oncogenesis were associated with the sensory perception of smell (154 out of 407 genes, q < 10^− 28^). Transient methylation during oncogenesis was associated with the mitotic cell cycle process (57 out of 777 genes, q = 0.012), and transient unmethylation was associated with post-transcriptional gene silencing (41 out of 615 genes, q = 0.0015) (Fig. 4, Additional File 1).

***A***: *Early hypomethylated genes*,* during the transition from non-tumor colonic tissue to low-grade adenoma and remain hypomethylated through high-grade adenoma and adenocarcinoma. These genes are involved in biological processes related to olfaction.* ***B***: *Early methylated genes*,* during the transition from non-tumor colonic tissue to low-grade adenoma and remain methylated through high-grade adenoma and adenocarcinoma. These genes are involved in biological processes related to nucleic acids process.* ***C***: *Transiently hypomethylated genes*,* undergo demethylation from non-tumor colonic tissue to adenoma*,* then become methylated again in adenocarcinoma. These genes are involved in biological processes related to post-transcriptional regulation.* ***D***: *Transiently methylated genes undergo demethylation from non-tumor colonic tissue to adenoma*,* then become methylated again in adenocarcinoma. These genes are involved in biological processes related to mitotic cell cycle.*

### Potential early colorectal DNA methylation biomarkers

#### Validated biomarkers are early

DNA methylation biomarkers for colorectal cancer, such as *SEPT9*, *NDRG4* and *BMP3*, which have received FDA approval for blood- or stool-based CRC screening, have been validated. Other biomarkers were tested after a review of published colorectal cancer biomarkers using DNA methylation in plasma or stool (Table 2).

**Table 2:** Colorectal cancer biomarkers described in the literature.

Gene	CpG	Location	Biomarker usage	Publication
*ADHFE1*	cg18065361	TSS200	stool	^27^
*BCAT1*	cg02765913	5UTR	plasma	^28^
*BMP3*	cg20276585	TSS200	plasma	^29^
*C9orf50*	cg18973112	TSS200	stool	^30^^31^,
*CLIP4*	cg09695033	TSS1500	stool	^32^
*CNRIP1*	cg11573679	1stExon	stool	^33^
*COL25A1*	cg07095995	TSS200	plasma	^34^
*FBN1*	cg15385562	TSS1500	stool	^35^
*FNB1*	cg15385562	TSS1500	stool	^33^
*FOXF1*	cg00314966	1stExon	plasma	^36^
*GATA5*	cg16714055	TSS1500	plasma	^37^
*GRIA4*	cg04747226	TSS200	stool	^38^
*HAND1*	cg03158581	TSS1500	plasma	^39^
*IKZF1*	cg23140175	TSS200	plasma	^28^
*KCNJ12*	cg27056599	TSS200	plasma	^31^
*KCNQ5*	cg09303936	TSS1500	stool	^30^
*LIFR*	cg11841722	TSS1500	plasma	^40^
*LINC00473*	cg09830769	TSS1500	plasma	^41^
*MAL*	cg04804539	TSS1500	stool	^33^
*METAP1D*	cg08750504	3UTR	plasma	^34^
*MPPED2*	cg11855526	5UTR	plasma	^42^
*NDRG4*	cg00687686	TSS1500	stool	^43^
*NPY*	cg00355281	TSS200	plasma	^44^
*PPP2R5C*	cg00723271	Body	stool	^27^
*SDC2*	cg24732574	TSS200	stool	^27^^43,45^,
*SEPT9*	cg17300544	TSS200	plasma	^46^
*SHOX2*	cg06759819	Body	plasma	^46^
*SNCA*	cg08767460	TSS1500	stool	^33^^35^,
*SPG20*	cg03966514	5UTR	stool	^33^
*TFPI2*	cg15649801	TSS1500	stool	^45,47^
*TWIST1*	cg09799658	TSS200	plasma	^31^
*VIPR2*	cg03976877	1stExon	stool	^38^
*WIF1*	cg26733786	5UTR	plasma	^44^
*ZNF132*	cg03735888	TSS200	plasma	^31^
*ZNF304*	cg21627760	TSS200	plasma	^40^

**Fig. 5:**
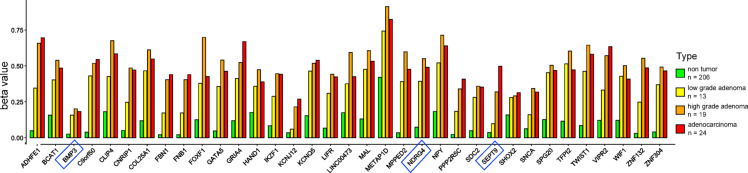
Methylation of known biomarkers.

An increase in methylation was observed for all known biomarkers (Fig. 5, Supplementary Table 2).

*BMP3* and *KCNJ12* were not methylated in adenocarcinomas. All the other known biomarkers were methylated in adenocarcinomas and in high-grade adenomas. *FBN1* and *SNCA* were not methylated in low-grade adenomas. The *SEPT9* and *VIPR2* promoters were not methylated in low-grade adenomas but were methylated in high-grade adenomas and adenocarcinomas.

*Potential and FDA-approved CRC biomarkers (marked with blue rectangles) based on DNA methylation in plasma and stools. The beta value cutoff used to determine methylation was 0.3. All of the biomarkers*,* except BMP3 and KCNJ12 are methylated during oncogenesis. ADHFE1*,* CNRIP1*,* FNB1*,* IKZF1*,* LIFR*,* PPP2R5C*,* SDC2*,* SEPT9*,* SHOX2*,* SNCA and ZNF132 are only partially methylated in low-grade adenomas and can be used to differentiate low-grade adenomas from high-grade adenomas or adenocarcinomas.*

### Comparison with an external dataset

A strong correlation was detected between the beta values of CpG sites from the two BeadChips (*n* = 452,034), with Spearman correlation coefficients consistently exceeding 0.9 (*p* < 10⁻⁴), as shown in Supplementary Fig. 2. Adenoma specific methylation changes were also observed in the dataset (Supplementary Fig. 3). Despite the high level of correlation, only 33% concordance was observed between the datasets. The biological processes associated with genes displaying methylation or unmethylation between non tumor colonic tissue and adenoma, and between adenoma and adenocarcinoma were consistent across both datasets (Additional File 2).

Two CpGs that were methylated in high-grade adenomas and adenocarcinomas, as well as two CpGs that were only methylated in adenocarcinomas, were tested using methylation-specific PCR and digital droplet PCR on non-tumor, adenoma, and adenocarcinoma samples from the Tumorothèque de Franche-Comté. The results confirmed the BeadChips data (*p* < 0.01 for each, Spearman’s test) (Supplementary Tables 3 and Supplementary Fig. 4).

## Discussion

To our knowledge, this study is the first to investigate the evolution of the colonic methylome in the context of distinguishing between low-grade and high-grade adenomas. Notably, we also report, for the first time, the presence of transient methylation changes during this progression. The 450k data revealed a global methylation gain during oncogenesis, whereas the EPIC data revealed a global methylation loss during oncogenesis. This inconsistent observation can be explained by the fact that the 450k focuses on gene promoters and that the EPIC BeadChip targets more intergenic CpGs, which are more affected by global hypomethylation in colorectal oncogenesis. The global methylation changes observed with the EPIC BeadChip are similar to those observed with immunohistochemistry^48^, especially concerning intergenic sequences such as long interspersed elements (LINEs).

We observed that the DNA methylation changes observed during colorectal oncogenesis were DNA evenly distributed between methylation and hypomethylation. The hypomethylation mostly occurs in CpGs in CpG shelves and open seas which has been previously described and associated with chromosomal instability in colorectal cancer^49^. Global DNA hypomethylation accounted for approximately 4% of the methylome, which is lower than the 8–10% reported via liquid chromatography^50^. These inconsistencies may be explained by the q value threshold used to determine the DMC (q < 10^− 4^) and by the fact that liquid chromatography is a quantitative measure that does not require the CpG to segregate in DMCs.

During oncogenesis, we noticed that the majority of DNA methylation modifications occurred during the transition between normal colon tissue and low-grade adenoma. This observation is inconsistent with the results of Andrew D Beggs et al.^11^, who used Illumina 27k BeadChips and reported that the DNA methylation pattern was acquired during the transition from adenoma to adenocarcinoma. However, our observation is consistent with the results of Yanxin Luo et al.^10^, who used the BeadChips Illumina 450k and reported that most of the methylation changes occur during the transition from normal colon mucosa to adenoma.

We found that global methylation clustering did not show a continuum from normal colon mucosa to adenocarcinoma but that the adenoma had its own methylation pattern. This effect can be observed in the results of Janssens et al.^14^. The magnitude of global methylation changes is the same, with approximately one-third of methylation and two-thirds hypomethylation occurring during the transition from non-tumor colon tissue to adenoma^10^.

Gene promoters with the earliest DMCs during oncogenesis were associated with tissular disorganization. The associated pathways are similar to those revealed by Lu YW et al.^12^, who used SeqCap-targeted bisulfite sequencing, and involve neuronal activity. This observation could be explained by the presence of mesenteric neurons in normal colon tissue, and an interesting phenomenon of tumor-neuron crosstalk could also explain these observations^51^. However, these explanations are not exclusive and need to be verified with additional studies.

The adenoma methylome confirmed the evolution of the methylation profile of 12 out of 15 described CRC biomarkers, and 10 out of 15 of these biomarkers occur early in oncogenesis, with differential methylation in low-grade adenomas, providing promising opportunities for DNA methylation CRC screening, diagnosis and follow-up. Biomarkers with methylation in high-grade adenomas may be relevant for the noninvasive assessment of tumor stage.

No batch effect was found between the two adenocarcinoma datasets (HCIM and GSE149282), but we cannot exclude a batch effect between the other datasets (Besançon adenoma and GSE132804). To limit potential batch effects, the methylation data were normalized. There are several ways to apply normalization to methylation BeadChip data: quantile normalization, Genome Studio normalization, SWAN normalization, functional normalization, and background and dye bias correction. Functional normalization combined with background and dye bias correction was chosen because it is the best fit when global changes are expected, such as in tumor versus non-tumor tissue comparisons, as in the case of this study. This method was developed for the 450k BeadChip and then adapted to the EPIC chip^52^.

Although computational normalization was performed uniformly to limit the batch effect, technical variability, such as differences in DNA extraction, bisulfite treatment, and array hybridization^53^, as well as biological variability such as genetic ancestry and sociocultural environment, may influence methylation profiles^54,55^. These factors should be considered when interpreting the results.

Future work may distinguish between different conventional colorectal adenoma subtypes, such as tubular, villous and tubulovillous adenomas based on methylation data. Additionally, exploring colorectal serrated lesions could provide new insight into the evolution of the colorectal methylome during oncogenesis. Our present study contains only a few numbers of samples, preventing a histological analysis. Samples with *MLH1* promoter methylation were excluded from the analysis, but those with MSI due to a mechanism other than *MLH1* promoter methylation could not be excluded.

The DMCs were not filtered by the absolute methylation difference due to the cellularity of adenoma samples; therefore, the DMCs were not expected to be quantitatively important, and an absolute methylation difference threshold would not be appropriate for the data. To limit the number of artifactual DMCs, a stringent FDR p value of 10^− 4^ was applied.

PCA did not identify subgroups of adenomas, but the small sample size is likely to have missed potential rare molecular subtypes of adenomas. The transcriptomic impact of methylation alterations was not investigated. The study of methylation coupled with transcriptomics is challenging due to the small amount of colorectal adenoma material and the high input required by these techniques.

## Conclusion

This study is the first to explore the dynamics of DNA methylation during the low-grade and high-grade adenoma stages of colorectal cancer oncogenesis and to suggest the potential reversibility of epigenetic alterations in this context. We examined the behavior of established DNA methylation biomarkers for colorectal cancer, providing insights into their potential utility for indirect tumor staging. Further research is required to validate these findings and assess their translational relevance.

## Supplementary Information

Below is the link to the electronic supplementary material.

Supplementary Material 1

Supplementary Material 2

Supplementary Material 3

## Data Availability

The methylome beta values and idat files of the adenomas from Besançon are available at the GEO accession number GSE288652. Other datasets generated and analyzed during this study are available from the corresponding author upon reasonable request.
