# Supplementary material for: Dynamic DNA methylation changes during colorectal oncogenesis with insights from adenoma stages
Source: Sci Rep. 2025 Nov 25;15:45283. doi: 10.1038/s41598-025-28656-5 (PMC12749741; doi:10.1038/s41598-025-28656-5)
Supplement: Supplementary file 3 — Supplementary Material 3 [file 41598_2025_28656_MOESM3_ESM.docx]

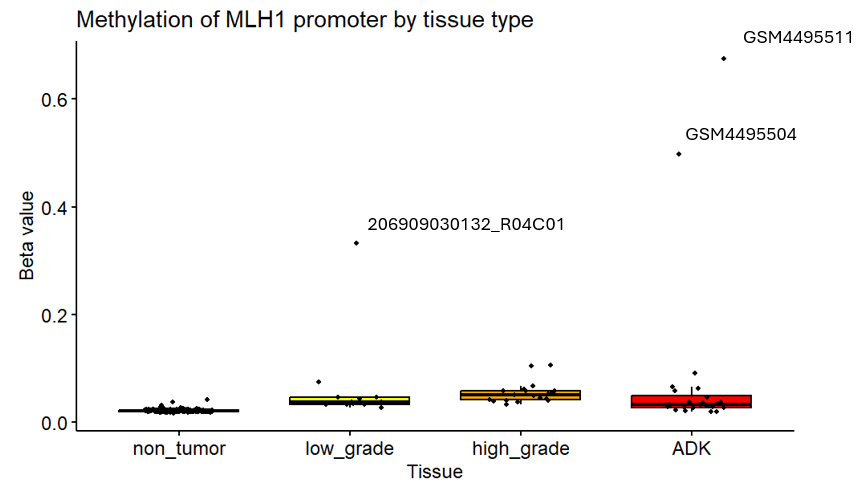


Supplementary figure 1 : Methylation of MLH1 promoter by tissue type

The MLH1 promoter methylation was defined as the mean of the probes in the CpG island in the 5’UTR of MLH1 (chr3:36992738-36993865 in hg38).

Supplementary table 1 : Methylation variation relative to CpG island

|  |  | CpGs in CpG island or Shore | CpGs in CpG Shelf or Open Sea | p value chi² |
| --- | --- | --- | --- | --- |
| Global changes | Methylation status not changed during oncogenesis | 268877 (85.1%) | 502466 (91.9%) | 2.20E-16 |
|  | Methylation status changed during oncogenesis | 47036 (14.9%) | 44548 (8.1%) |  |
| Definitive methylation | Methylation at low-grade adenoma | 29931 (63.6%) | 2741 (6.2%) | 2.2E-16 |
|  | Methylation at high-grade adenoma | 4844 (10.3%) | 473 (1.1%) |  |
|  | Methylation at adenocarcinoma | 2182 (4.6%) | 1540 (3.5%) |  |
| Definitive hypomethylation | hypomethylation at low-grade adenoma | 5030 (10.7%) | 24090 (54.1%) | 2.2E-16 |
|  | hypomethylation at high-grade adenoma | 885 (1.9%) | 3515 (7.9%) |  |
|  | hypomethylation at adenocarcinoma | 889 (1.9%) | 7258 (16.3%) |  |
| Transitory methylation | transitory methylation in low-grade adenoma | 95 (0.2%) | 49 (0.1%) | 2.2E-16 |
|  | transitory methylation in high-grade adenoma | 249 (0.5%) | 37 (0.1%) |  |
|  | transitory methylation in low-grade and high-grade adenoma | 739 (1.6%) | 92 (0.2%) |  |
| Transitory hypomethylation | transitory hypomethylation in low-grade adenoma | 217 (0.5%) | 831 (1.9%) | 2.2E-16 |
|  | transitory hypomethylation in high-grade adenoma | 471 (1%) | 1056 (2.4%) |  |
|  | transitory hypomethylation in low-grade adenoma and high-grade adenoma | 1504 (3.2%) | 2866 (6.4%) |  |

Supplementary Table 2 : Methylation mean of known biomarkers

| gene | cg | location | non tumor colorectal tissue | low grade adenoma | high grade adenoma | adenocarcinoma |
| --- | --- | --- | --- | --- | --- | --- |
| ADHFE1 | cg18065361 | TSS200 | 0.05 | 0.34 | 0.66 | 0.70 |
| BCAT1 | cg02765913 | 5UTR | 0.16 | 0.40 | 0.54 | 0.48 |
| BMP3 | cg20276585 | TSS200 | 0.03 | 0.16 | 0.20 | 0.18 |
| C9orf50 | cg18973112 | TSS200 | 0.04 | 0.43 | 0.52 | 0.54 |
| CLIP4 | cg09695033 | TSS1500 | 0.18 | 0.43 | 0.68 | 0.58 |
| CNRIP1 | cg11573679 | 1stExon | 0.05 | 0.25 | 0.49 | 0.47 |
| COL25A1 | cg07095995 | TSS200 | 0.12 | 0.47 | 0.61 | 0.55 |
| FBN1 | cg15385562 | TSS1500 | 0.02 | 0.17 | 0.40 | 0.44 |
| FNB1 | cg15385562 | TSS1500 | 0.02 | 0.17 | 0.40 | 0.44 |
| FOXF1 | cg00314966 | 1stExon | 0.13 | 0.38 | 0.70 | 0.43 |
| GATA5 | cg16714055 | TSS1500 | 0.05 | 0.36 | 0.54 | 0.46 |
| GRIA4 | cg04747226 | TSS200 | 0.12 | 0.41 | 0.52 | 0.67 |
| HAND1 | cg03158581 | TSS1500 | 0.18 | 0.36 | 0.47 | 0.39 |
| IKZF1 | cg23140175 | TSS200 | 0.08 | 0.29 | 0.45 | 0.44 |
| KCNJ12 | cg27056599 | TSS200 | 0.03 | 0.06 | 0.21 | 0.27 |
| KCNQ5 | cg09303936 | TSS1500 | 0.15 | 0.46 | 0.52 | 0.54 |
| LIFR | cg11841722 | TSS1500 | 0.07 | 0.31 | 0.44 | 0.42 |
| LINC00473 | cg09830769 | TSS1500 | 0.17 | 0.38 | 0.59 | 0.43 |
| MAL | cg04804539 | TSS1500 | 0.13 | 0.48 | 0.61 | 0.53 |
| METAP1D | cg08750504 | 3UTR | 0.42 | 0.74 | 0.91 | 0.82 |
| MPPED2 | cg11855526 | 5UTR | 0.03 | 0.39 | 0.60 | 0.48 |
| NDRG4 | cg00687686 | TSS1500 | 0.07 | 0.39 | 0.55 | 0.49 |
| NPY | cg00355281 | TSS200 | 0.18 | 0.52 | 0.71 | 0.64 |
| OPLAH | cg26256223 | Body | 0.05 | 0.55 | 0.75 | 0.72 |
| PPP2R5C | cg00723271 | Body | 0.02 | 0.18 | 0.34 | 0.41 |
| SDC2 | cg24732574 | TSS200 | 0.05 | 0.28 | 0.36 | 0.35 |
| SEPT9 | cg17300544 | TSS200 | 0.04 | 0.10 | 0.32 | 0.50 |
| SHOX2 | cg06759819 | Body | 0.16 | 0.28 | 0.29 | 0.31 |
| SNCA | cg08767460 | TSS1500 | 0.06 | 0.16 | 0.34 | 0.32 |
| SPG20 | cg03966514 | 5UTR | 0.13 | 0.45 | 0.50 | 0.47 |
| TFPI2 | cg15649801 | TSS1500 | 0.12 | 0.51 | 0.60 | 0.47 |
| TWIST1 | cg09799658 | TSS200 | 0.08 | 0.46 | 0.64 | 0.58 |
| VIPR2 | cg03976877 | 1stExon | 0.12 | 0.33 | 0.57 | 0.63 |
| WIF1 | cg26733786 | 5UTR | 0.12 | 0.43 | 0.50 | 0.41 |
| ZNF132 | cg03735888 | TSS200 | 0.03 | 0.25 | 0.55 | 0.49 |
| ZNF304 | cg21627760 | TSS200 | 0.04 | 0.37 | 0.49 | 0.47 |


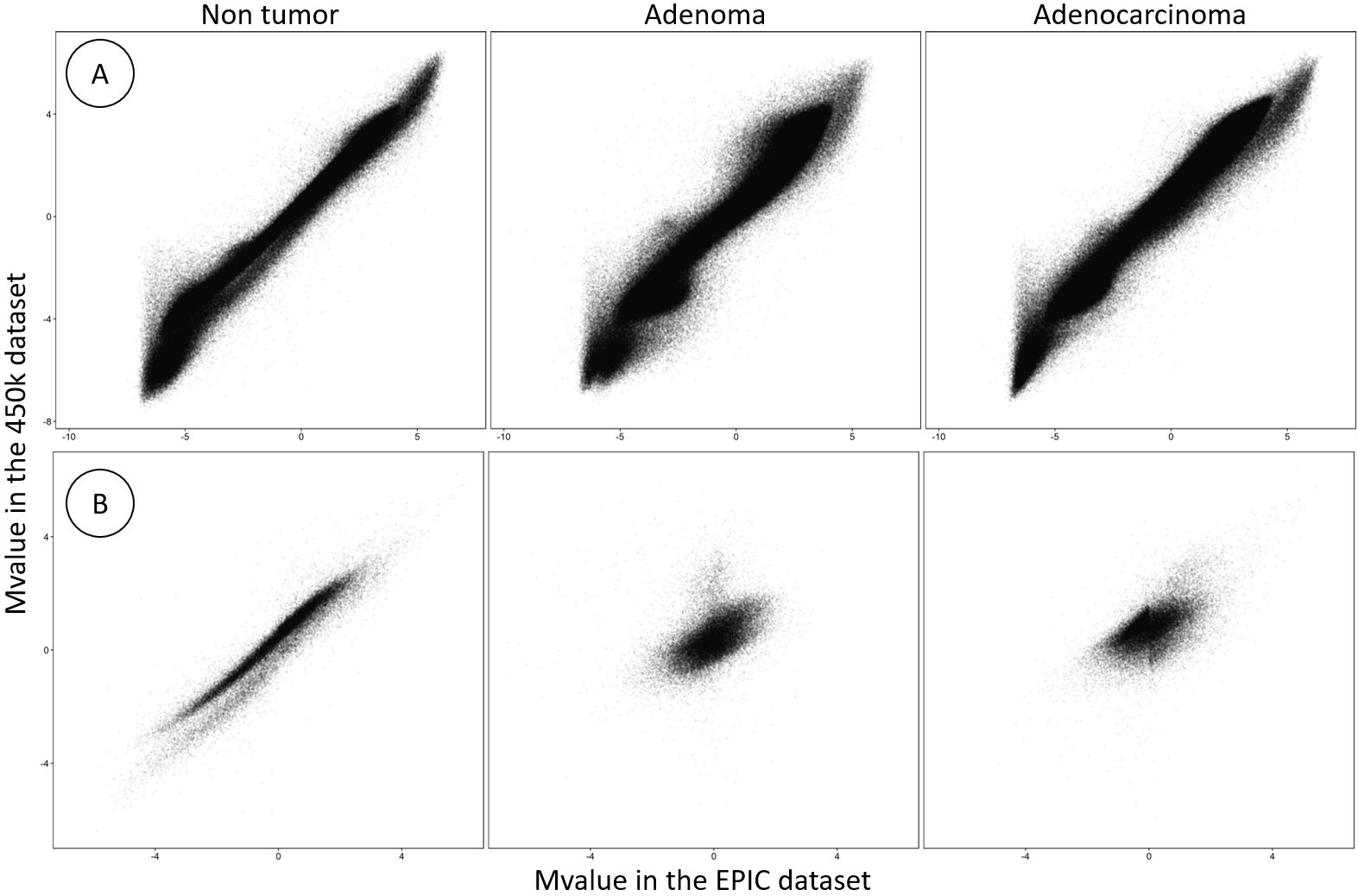


Supplementary figure 2 : (A) correlation of the methylation of all cpg between the two datasets. (B) correlation of the methylation of the CpG differentially methylated in the two datasets.


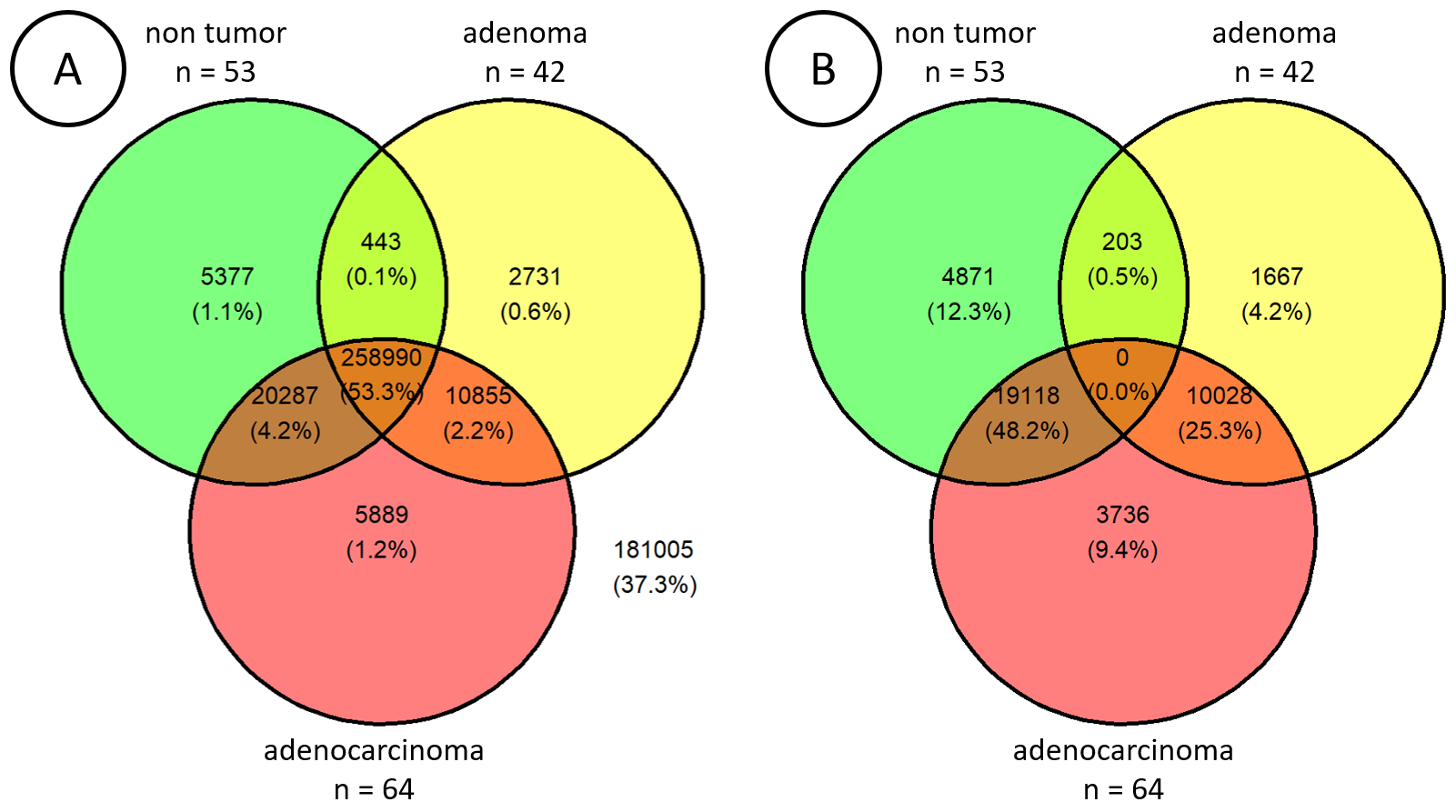


Supplementary figure 3 : Venn diagram of DNA methylation in the 450k dataset

A: Venn diagram of the methylation status of the CpG on autosomes and without problematic probes shared between the 450k and EPIC Beadchip across the sample types in the GEO datasets GSE48684 and GSE42921 that use the 450k Beadchip. B: Distribution of CpG differentially methylated with student test after FDR correction (q < 10-4).

Supplementary table 3 : Primers used for the digital methylation specific PCR.

| Methylation on Beadchip data | Illumina CpG ID | Position (hg38) | Gene - position | Forward primer | Reverse primer |
| --- | --- | --- | --- | --- | --- |
| Methylated in HG* and ADK** | cg21938148 | chr13:110306629 | COL4A1 – Gene body | GAC-GGG-TGA-AGG-CGT-TTA-GTT-GTC | AAA-AAA-CAA-CCC-GCG-CTA-TAA-CGA-A |
| Methylated in HG* and ADK** | cg16601494 | chr1:1540356 | C1orf70 – 5’UTR | GTT-TAT-GGA-TAT-CGG-GCG-GGG-TC | AAA-ACG-ACG-AAA-ACT-CAA-CAT-CGA-A |
| Methylated in HG* and ADK** | cg24033330 | chr20:40688393 | MAFB – 1^st^ exon | AGT-TCG-CGT-ACG-GAT-ATG-GAT-ACG-A | GAC-AAC-GAC-AAC-GTA-AAA-AAC-CGC-T |
| Methylated in HG* and ADK** | cg08808128 | chr2:29115565 | CLIP4 – 1^st^ exon | TTA-TCG-AGT-TTT-TAG-CGC-GTGCGC | ACC-TCT-CCG-AAC-CCC-TCC-CG |
| Independent of methylation | . | chr20:19270743 | SLC24A1 – 1^st^ intron | TTG-TAT-GTA-TGT-GAG-TGT-GGG-AGA-GAG-A | TTT-CTT-CCA-CCC-CTT-CTC-TTC-C |

* HG : High-grade adenoma ** ADK : Adenocarcinoma

The digital methylation specific PCR was performed with the QX200 ddPCR EvaGreen Supermix on the QX200 system (Biorad, USA), according to the manufacturer’s instructions. The methylation percentage was calculated by dividing the copy number of the methylated primer by the copy number of the C-Less primer.


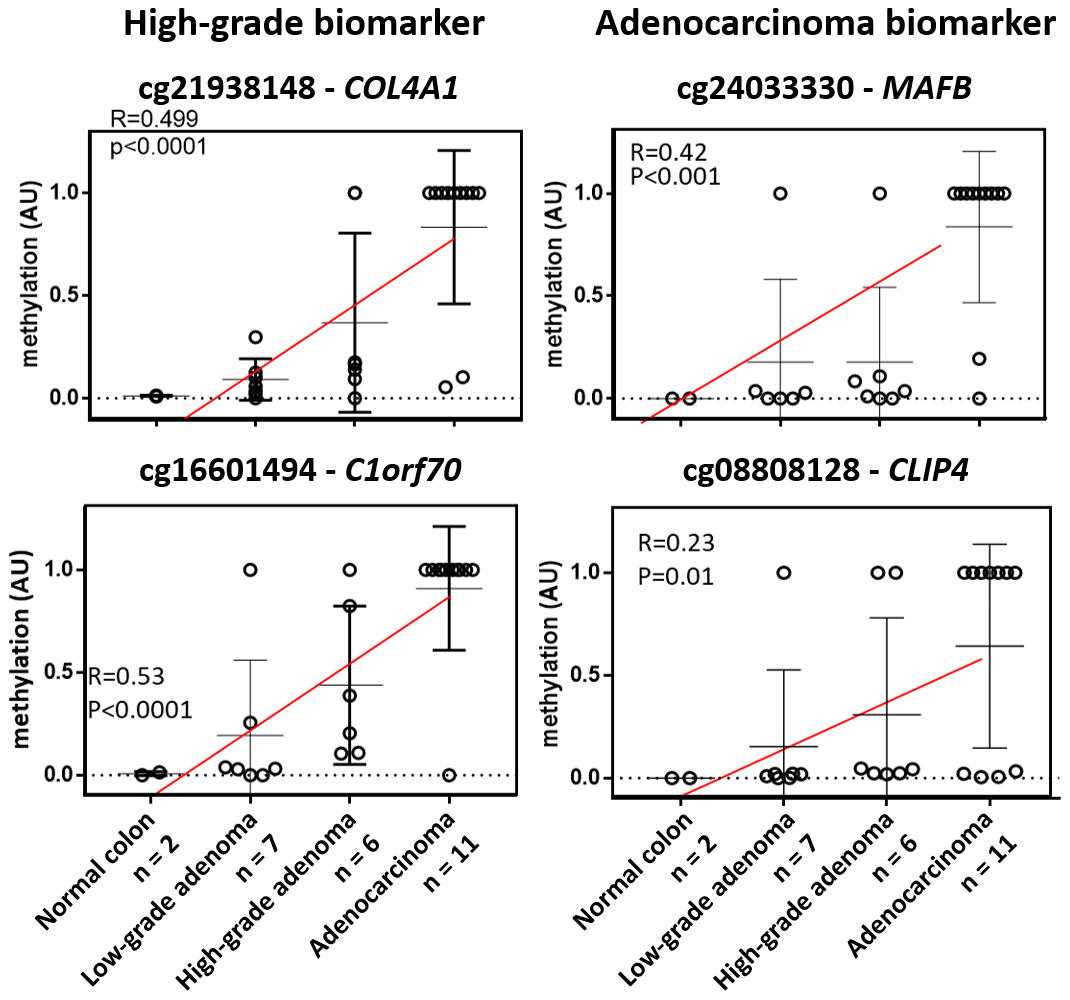


Supplementary figure 4 : Methylation-specific PCR and digital droplet PCR on normal colonic tissues, low-grade adenomas, high-grade adenomas, and adenocarcinomas.

The selected CpGs showed increased methylation according to tumor stage (p < 0.01, Spearman's test). CpGs cg21938148 and cg16601494 showed increased methylation in high-grade adenomas and adenocarcinomas, while CpGs cg24033330 and cg08808128 showed increased methylation only in adenocarcinomas.
